# Novel major QTLs associated with low soil phosphorus tolerance identified from the Indian rice landrace, Wazuhophek

**DOI:** 10.1371/journal.pone.0254526

**Published:** 2021-07-15

**Authors:** Ravindra Ramrao Kale, Ch. V. Durga Rani, M. Anila, H. K. Mahadeva Swamy, V. P. Bhadana, P. Senguttuvel, D. Subrahmanyam, M. Ayyappa Dass, K. Swapnil, M. S. Anantha, E. Punniakotti, B. Laxmi Prasanna, G. Rekha, P. Sinha, M. B. V. N. Kousik, T. Dilip, S. K. Hajira, P. Brajendra, S. K. Mangrauthia, C. Gireesh, Mangaldeep Tuti, R. Mahendrakumar, Jitendra Giri, Pawandeep Singh, R. M. Sundaram

**Affiliations:** 1 Institute of Biotechnology, The Professor Jayashankar Telangana State Agricultural University, Rajendranagar, Hyderabad, India; 2 Indian Council of Agricultural Research -Indian Institute of Rice Research, Rajendranagar, Hyderabad, India; 3 Indian Council of Agricultural Research—Sugarcane Breeding Institute, Coimbatore, India; 4 Indian Council of Agricultural Research -Indian Institute of Agricultural Biotechnology, Ranchi, India; 5 Department of Biotechnology - National Institute of Plant Genome Research, New Delhi, India; National Institute for Plant Genome Research, INDIA

## Abstract

With an objective of mapping novel low soil P (Phosphorus) tolerance loci in the non-*Pup1* type donor rice line, Wazuhophek, we screened a recombinant inbred line (RIL) mapping population consisting of 330 lines derived from the cross Wazuhophek x Improved Samba Mahsuri (which is highly sensitive to low soil P) in a plot with low soil P for tolerance associated traits. Molecular mapping with SSR markers revealed a total of 16 QTLs (seven major and nine minor QTLs), which are associated with low soil P tolerance related traits. Interestingly, a QTL hotspot, harbouring 10 out of 16 QTLs were identified on the short arm of chromosome 8 (flanked by the makers RM22554 and RM80005). Five major QTLs explaining phenotypic variance to an extent of 15.28%, 17.25%, 21.84%, 20.23%, and 18.50%, associated with the traits, plant height, shoot length, the number of productive tillers, panicle length and yield, respectively, were located in the hotspot. Two major QTLs located on chromosome 1, associated with the traits, total biomass and root to shoot ratio, explaining 15.44% and 15.44% phenotypic variance, respectively were also identified. Complex epistatic interactions were observed among the traits, grain yield per plant, days to 50% flowering, dry shoot weight, and P content of the seed. *In-silico* analysis of genomic regions flanking the major QTLs revealed the presence of key putative candidate genes, possibly associated with tolerance.

## Introduction

Rice is one of the world’s most important and principal cereal crops and more than half of the global population derive their calorific requirements from it. Reduced availability of water and arable land, the constant threat of biotic and abiotic stresses and adverse effects of a rapidly changing climate, nutrient-deficient soils, etc. are some of the major threats to rice production and food security [1]. Phosphorous (P) is an essential nutrient for all living organisms including rice. P deficiency in soil is one of the major limitations of rice production as it significantly affects rice growth and development in terms of reduced plant growth, reduced root development, lesser number of productive tillers, higher spikelet sterility, delayed flowering and increased root elongation [2, 3]. It is predicted that the annual increase in P fertilizer consumption will be 20 million tons by 2030 and the P fertilizer sources are finite [4]. Cultivated plants use only 20–30% of the applied phosphorus-fertilisers, while rest is rapidly immobilized owing to fixation and microbial activity [5]. In acidic soils, free iron and aluminium oxides bind to native and applied P, whereas in calcareous soils, the abundant calcium and magnesium compounds bind inorganic phosphates (Pi) making it unavailable to plants [6]. Plants cope with low P in the soil by developing adaptive changes at morphological, physiological, biochemical and molecular levels for enhanced Pi uptake or increased internal P-use-efficiency for which basic mechanisms are P-acquisition-efficiency (P uptake) and internal P-use-efficiency (PUE). Low P tolerance is a complex quantitative trait [7]. Fortunately, rice germplasm have significant variations for the traits related to low soil P tolerance and associated adaptive mechanisms of rice plant to low-Pi stress, thus allowing the designing of effective breeding and genetic engineering strategies to produce low P tolerant or P use efficient rice lines [8]. A major QTL associated with low soil P tolerance, named *Pup1* explaining upto 80% phenotypic variation, was identified earlier on chromosome12 of the Indian rice landrace, Kasalath [9]. Later on *Pup1* was fine-mapped and the causative gene underlying *Pup1* was identified as, *OsPSTOL1* (encoding a protein kinase) [10]. In search of novel sources of low soil P tolerance (i.e. other than *Pup1*), we earlier carried out the genotypic and phenotypic screening of Indian rice germplasm for low soil P. This effort culminated in the identification of a rice line from North-East part of India, named Wazuhophek, which showed excellent tolerance to low soil P and was devoid of *Pup1* [3]. We designed and carried out this study intending to establish the genetic basis of tolerance to low soil P in Wazuhophek and undertake molecular mapping of tolerance associated loci using a recombinant inbred line mapping population developed from the cross between Wazuhophek (low soil P tolerant) and Improved Samba Mahsuri (low soil P sensitive).

## Material and methods

### Plant materials

Wazuhophek, a landrace from North-Eastern part of India, which has been reported to possess excellent tolerance to low soil P and is devoid of the well-known low soil P tolerance QTL, *Pup1* [3] was used as one of the parents for crossing with Improved Samba Mahsuri (ISM), a low soil P sensitive rice variety. ISM is a high-yielding, fine-grain type rice variety, possessing low glycemic index (GI), developed through the process of marker-assisted backcross breeding and possesses three major bacterial blight resistance genes viz., *Xa21*, *xa13*, and *xa5* in the genetic background of the Indian mega-variety of rice, Samba Mahsuri [11]. A recombinant inbred line (RIL) population consisting of 330 lines was developed at ICAR-Indian Institute of Rice Research (ICAR-IIRR), Hyderabad, by advancing the F_2_ plants derived from the cross Wazuhophek and ISM through single seed descent (SSD) method. A sub-set of the above said population consisting of 112 RILs (at F_6_ generation) was used for molecular mapping of the loci associated with low P tolerance traits in Wazuhophek.

### Evaluation of mapping population for low P tolerance and agro-morphological traits

Seedlings were raised using wet bed nursery. The 21 days old seedlings of RILs were transplanted in the low P soil plot (available Phosphorus < 2 kg ha^-1^) [12] along with the donor and recipient parents and the check variety, Swarna, a low soil P tolerant line, possessing *Pup1* [12] for evaluation of agro-morphological traits [3]. The experiment was conducted in RBD with three replications, the seedlings were planted by spacing of 20 X 15 cm, plants were applied with basal application of N, K, Fe and Zn followed by top dressing N at maximum tillering stage, and no phosphatic fertilizer was applied during the entire crop season. The available soil P levels were monitored during transplanting, maximum tillering and grain maturity stages. Observations were recorded for traits affected by low soil P like days to 50% flowering (DFF), plant height (PH), number of productive tillers (NPT), panicle length (PL), shoot length (SL), 1000 grain weight, grain yield per plant, P content in grains and traits helpful in imparting low soil P tolerance like root growth, root volume, root to shoot ratio, biomass were recorded [3]. Three plants were selected randomly which represent the entire line and mean data was subjected to statistical analysis using SAS 9.2 (SAS version 9.2 software package (SAS Institute, Inc.; Cary, NC). Correlation among the agro-morphological traits was analysed using open-source statistical software ’R’ version 3.6.3 (R Core Team, 2016).

### DNA isolation and genotyping

DNA was isolated from the parents and the RILs using the CTAB protocol [13]. A set of 587 hyper-variable rice microsatellite markers (i.e. rice SSR markers; details available at https://archive.gramene.org/markers/microsat/all-ssr.html) were used to check polymorphism between donor parent, Wazuhophek and recipient parent ISM. Out which a total of 78 parental polymorphic SSR markers, which are distributed evenly across the rice genome were selected and used for molecular mapping. PCR reactions were performed in a final volume of 20μl [3].

### Mapping of genomic regions associated with low soil P tolerance to identify closely linked genes/QTLs and data analysis

Linkage analysis and map construction was performed using the genotype data derived from 78 parental polymorphic SSR markers distributed across the 12 chromosomes of rice (with an average distance of ~ 5 Mb between the markers) along with the phenotype data derived from the selected 112 RILs under low soil P condition, using the software program QTL IciMapping version 4.0 [14]. The map distances were converted into cM, using the Kosambi function [15]. Three steps, namely grouping, ordering, and rippling was followed in order, after which output command was given and the complete linkage map was obtained. QTL analysis was performed using ICIM (inclusive composite interval mapping) for molecular mapping of the low soil P tolerance associated loci. LOD score of 2.5 was set as a threshold for the identification of QTLs with significant effects. Nomenclature for QTL was followed as described earlier [16]. A QTL contributing more than 15% of the total phenotypic variance on the trait was considered as major effect QTL [17].

### *In-silico* identification of putative candidate genes associated with low soil P tolerance using mapping data

To analyse the putative candidate genes present in the chromosomal regions associated with low soil P tolerance, information from the genomic intervals underlying the major QTLs identified in this study was used for *in-silico* analysis with the databases, viz., RAP-DB (https://rapdb.dna.affrc.go.jp/), QTARO database (http://qtaro.abr.affrc.go.jp/) and Rice genome annotation project (http://rice.plantbiology.msu.edu/). The genes were grouped according to their annotation.

## Results

### Phenotypic variations for low soil P tolerance in the parents and RILs

**Fig 1:**
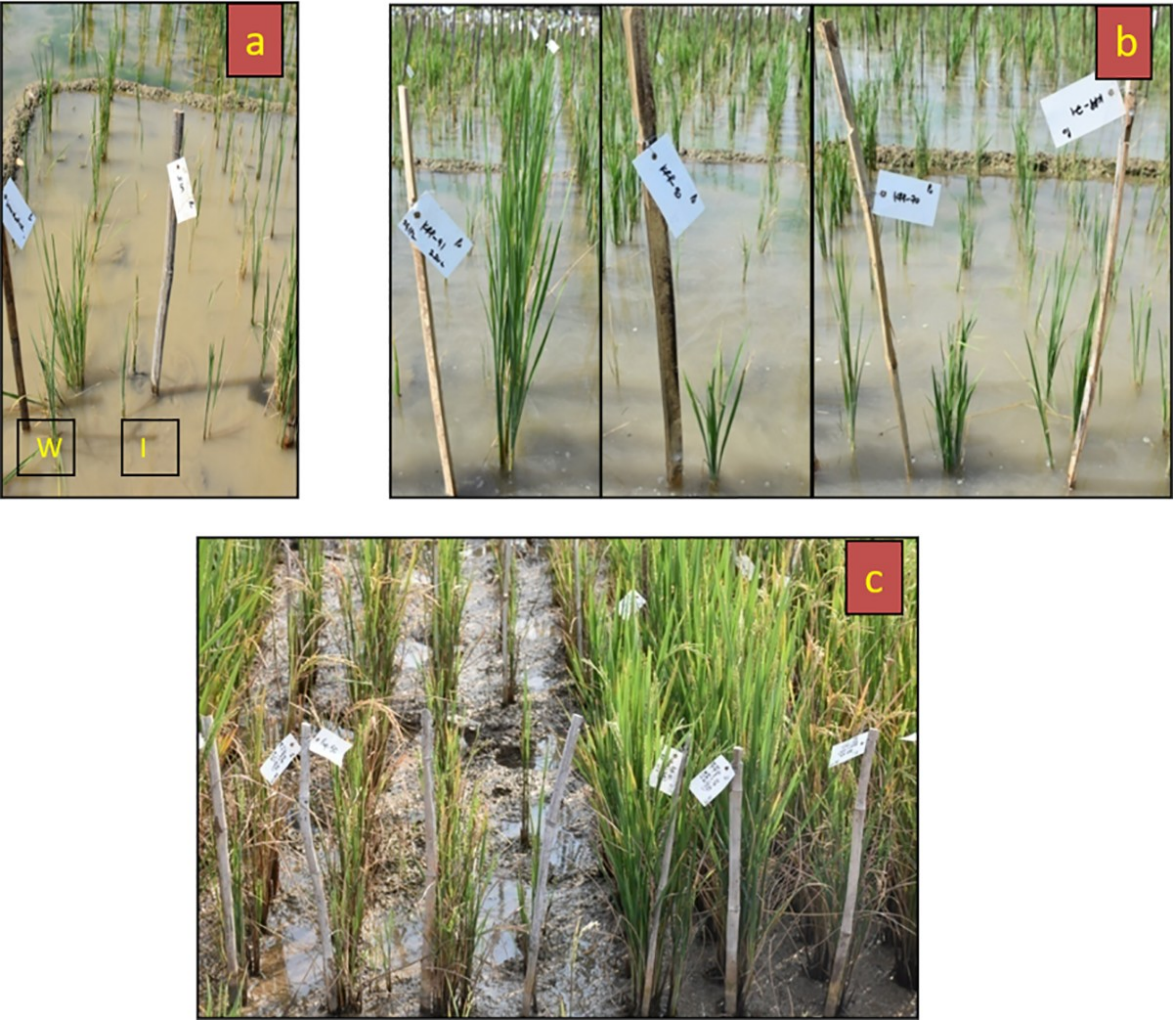
Screening of recombinant inbred line (RIL) population derived from the cross Wazuhophek x Improved Samba Mahsuri in low soil P plot at ICAR- IIRR, Hyderabad. a- Response of parents, W (Wazuhophek) and I (ISM). Wazuhophek shows high level of tolerance, while ISM is highly sensitive in the low soil P plot. b- RILs at vegetative stage showing variation for tolerance in the low soil P plot (i.e. from highly sensitive to good level of tolerance). c- Performance of individual RILs at flowering stage in the low soil P plot. While some lines are showing high sensitivity, others are clearly showing tolerance in low soil P conditions.

**Fig 2:**
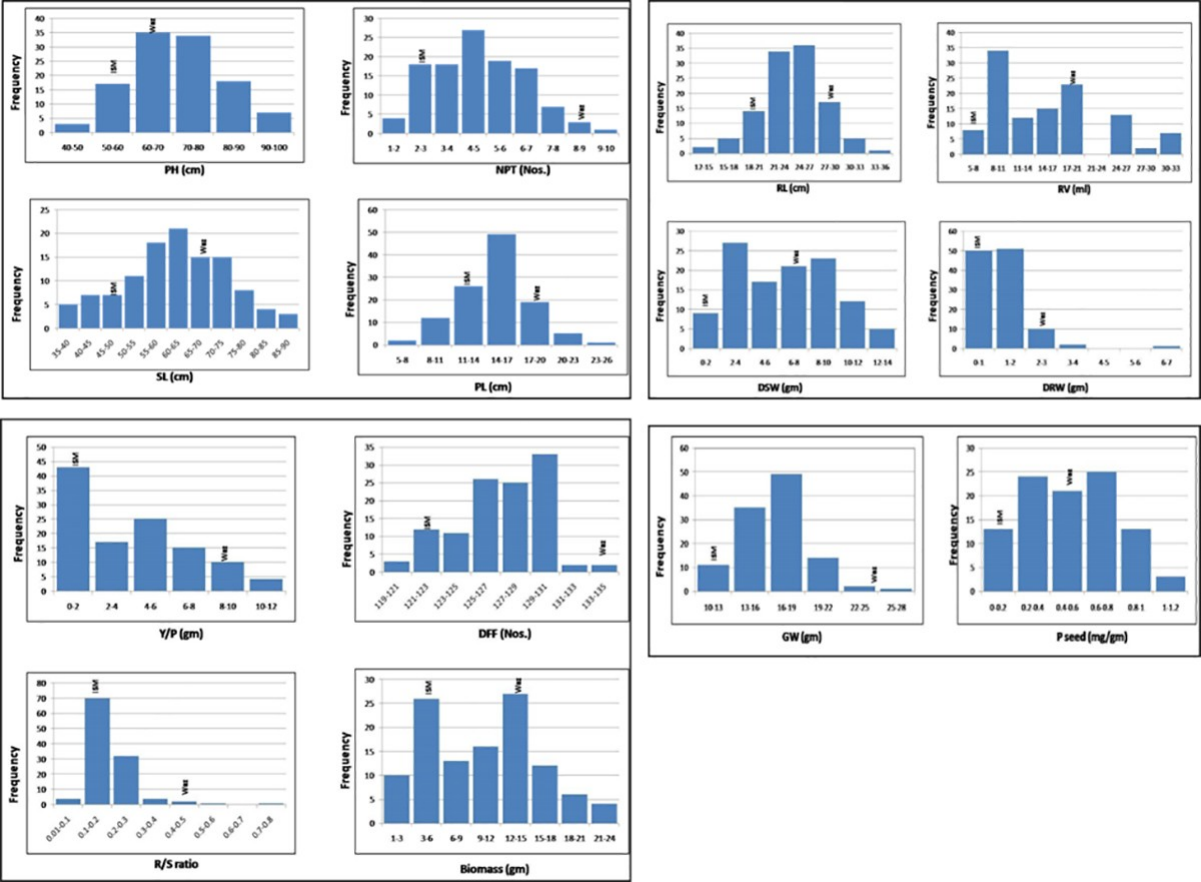
Phenotypic distribution shown by the RILs for various low soil P related traits in the low soil P plot at ICAR-IIRR. Waz- Wazuhophek, ISM- improved samba Mahsuri, PH- plant height, NPT- number of productive tillers, SL- shoot length, PL- panicle length, RL- root length, RV- root volume, DSW- dry shoot weight, DRW- dry root weight, Y/P- yield per plant, DFF- days to fifty percent flowering, R/S ratio- root to shoot ratio, GW- 1000 grain weight, P seed- P content in seed.

The RIL population showed continuous variation (i.e. sensitive, moderately tolerant, and highly tolerant responses) for all the traits studied in low soil P condition (Fig 1). Significant variations were observed for most of the morphological traits with significant delay in flowering (Fig 2). The details of mean, range, standard deviation, transgressive segregation, Kurtosis and Skewness for all the traits in RILs under low soil P is given in S1 Table. Negative skewness was observed for shoot length, panicle length, root length, and days to 50% flowering. However, the RILs showed positive skewness for plant height, number of effective tillers per plant, dry shoot weight, dry root weight, root volume, root to shoot ratio, yield per plant, 1000 seed weight, total biomass, and P content in seeds. Maximum skewness was observed for root to shoot ratio, whereas minimum skewness was observed for days to 50% flowering. All the other traits were positively skewed with maximum skewness for number of effective tillers per plant and minimum skewness observed for shoot length. The transgressive segregants ranged from 5.26% (for root to shoot ratio) to 67.54% (for dry shoot weight). The nature of the relationship among 14 quantitative characters under low soil P condition in the RILs and their parents was determined through correlation analysis. A total of 91 combinations were formed for fourteen traits under consideration. Among these, 65 combinations were found to be significant and positively correlated (Table 1). Most of the traits were positively associated with each other except for root to shoot ratio and days to fifty percent flowering, which showed negative and non-significant association with most of the traits under study. In particular, the trait, panicle length was found to be strongly correlated with shoot length and plant height. Further, yield per plant was found to be closely associated with shoot length and number of productive tillers. Biomass showed strong correlation with shoot length, dry shoot weight, and yield per plant. Based on the results obtained, these traits could be targets for selection in breeding programs aimed at improvement of low soil P tolerance in rice.

**Table 1:** Values of the correlation coefficients between low soil P tolerance related agro-morphological traits of in RILs grown under low soil P condition.

	PH	SL	NPT	PL	RL	RV	DSW	DRW	SY	1000sw	DFF	Bio	RS ratio	P seed
PH	1													
SL	0.9	1												
NPT	0.48	0.56	1											
PL	0.78	0.81	0.66	1										
RL	0.5	0.56	0.36	0.44	1									
RV	0.54	0.61	0.42	0.43	0.65	1								
DSW	0.66	0.73	0.45	0.52	0.62	0.69	1							
DRW	0.41	0.48	0.37	0.38	0.41	0.48	0.51	1						
SY	0.64	0.72	0.71	0.66	0.4	0.51	0.55	0.39	1					
1000sw	0.49	0.53	0.37	0.46	0.29	0.28	0.39	0.32	0.57	1				
DFF	-0.17	-0.21	-0.07	-0.17	-0.17	-0.11	-0.13	-0.08	-0.17	-0.16	1			
Bio	0.74	-0.82	0.65	0.67	0.58	0.69	0.89	0.52	0.87	0.54	-0.17	1		
RS ratio	-0.01	-0.02	0.08	0.02	0	-0.05	-0.14	0.55	0.01	0.09	-0.13	-0.07	1	
P seed	0.5	0.58	0.49	0.48	0.38	0.42	0.62	0.3	0.61	0.47	-0.09	0.7	-0.06	1

Note:- PH- Plant height, SL- Shoot length, NPT- Number of productive tillers, PL- Panicle length, RL- Root length, RV- Root volume, DSW- Dry shoot weight, DRW-Dry root weight, SY- Yield per plant, 1000sw- Thousand seed weight, DFF- days to fifty percent flowering, Bio- Total biomass, RS ratio- root to shoot ratio, P seed- Phosphorus content in seed.

### Identification of QTLs for the low soil P tolerance

**Fig 3:**
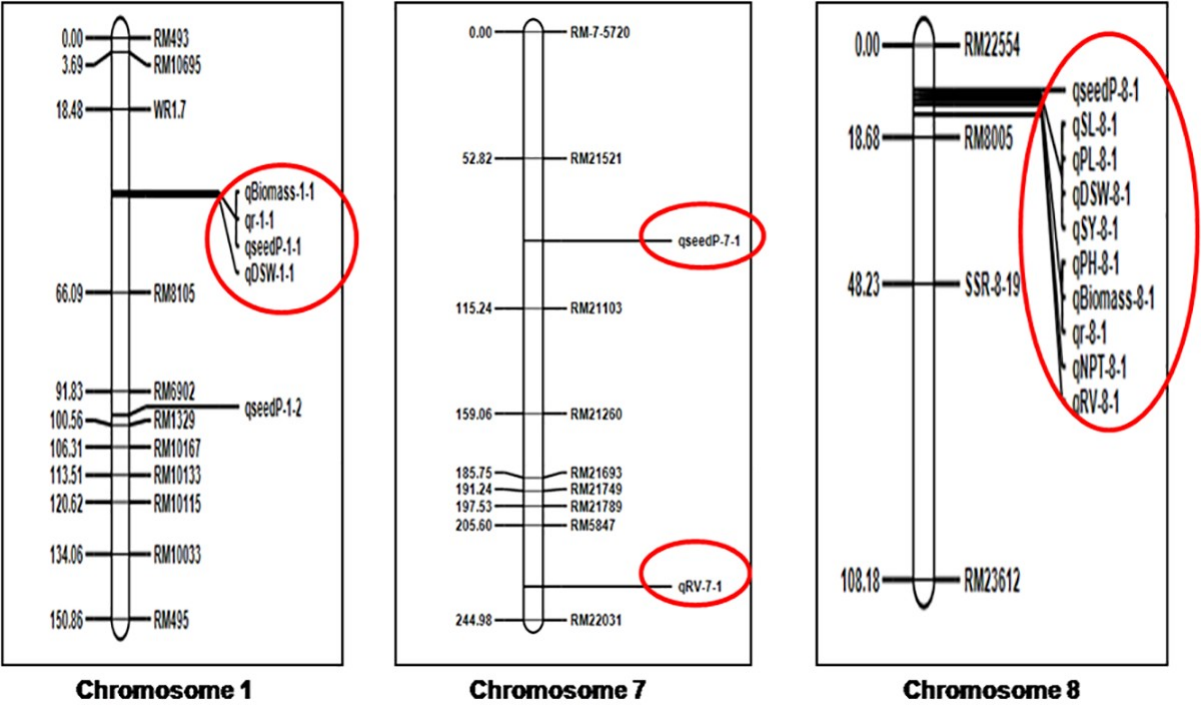
Linkage map and the position of identified QTLs for low soil P tolerance. qPH- Plant height, qSL- Shoot length, qNPT- Number of productive tillers, qPL- Panicle length, DSW- Dry shoot weight, RV- Root volume, qSY- Yield per plant, qBiomass- Total biomass, qseedP- Phosphorus content of seed. Major QTL hot-spots were noticed on Chr. 1 and Chr. 8.

The phenotypic and genotypic data collected from the RILs was used to construct a linkage map and carry out QTL analysis. The SSR markers covered a total length of 1633.05 cM across the 12 chromosomes with an average distance of 20.93 cM between the markers. An SSR linkage map was then constructed and used for QTL mapping (S1 Fig and Fig 3; Table 2). The region between the marker interval, WR1.7- RM8105 on chromosome 1 encompassed four QTLs for the traits- dry shoot weight (*qDSW1*), total biomass (*qBiomass1*), root to shoot ratio (*qr1*) and phosphorus content in seed (*qseedP1*), with LOD scores ranging from 2.75 to 3.24 and the phenotype variance ranging from 10.51 to 15.44%. Two QTLs were detected on chromosome 7, one of these QTLs was associated with root volume (*qRV7*) and was localized between the markers, RM5847-RM22031 with a LOD score of 3.05 and explaining 14.33% of the phenotype variance. The other QTL on chromosome 7 was for P content in seed (denoted as *qSP7*) and was located between the markers, RM21521-RM21103 with a LOD score of 2.87, accounting for 10.01% to the phenotypic variation. Most of the major QTLs, including those for yield and other key agro-morphological traits, were located on chromosome 8, between the markers, RM22554 and RM8005. Thus, this region may be considered as a QTL hot-spot. A total of ten QTLs were detected in this marker interval for different traits related to low soil P tolerance, such as plant height (*qPH-8*), shoot length (*qSL-8*), number of productive tillers (*qNPT-8*), panicle length (*qPL-8*), grain yield per plant, dry shoot weight (*qDSW-8*), root volume (*qRV-8*), total biomass (*qBiomass-8*), root to shoot ratio (*qr-8*), P content of seed (*qseedP-8*) with LOD score ranging from 2.75 to 6.02, contributing 2.25 to 21.84% of phenotype variance. Out of seven major QTLs identified in the current study, five were located on chromosome 8 in the above mentioned marker interval and they were associated with the traits- plant height (denoted as *qPH-8*) [LOD: 4.22 and PVE: 15.28%], shoot length (*qSL-8*) [LOD: 3.32 and PVE: 17.25%], number of productive tillers (*qNPT-8*) [LOD: 6.02 and PVE: 21.84%], panicle length (*qPL-8*) [LOD: 5.97 and PVE: 20.23%] and grain yield per plant (*qSY-8*) [LOD: 5.50 and PVE: 18.50%]. The other two major QTLs were identified on chromosome 1 for biomass (*qBiomass-1*) [LOD: 3.10 and PVE: 15.44%] and root to shoot ratio (qr) [LOD: 3.10 and PVE: 15.44%].

**Table 2:** QTL(s) detected for low soil P tolerance in RIL population of Wazuhophek x Improved Samba Mahsuri.

SI. no	Trait	Chr. No.	Associated markers	LOD	PVE (%)	Add	Position (cM)	Position (Mb)	Size (Mb)
1	Plant height (PH)	8	RM22554(L)-RM8005(R)	4.22	15.28	5.83	11	5.5–1.01	4.49
2	Shoot length (SL)	8	RM22554(L)- RM8005(R)	3.32	17.25	4.49	10	5.5–1.01	4.49
3	Number of productive tillers (NPT)	8	RM22554(L)- RM8005(R)	6.02	21.84	0.96	12	5.5–1.01	4.49
4	Panicle length (PL)	8	RM22554(L)- RM8005(R)	5.97	20.23	1.79	10	5.5–1.01	4.49
5	Dry shoot weight (DSW)	1	WR 1.7(L)-RM8105(R)	3.24	14.11	2.34	41	1.7–10.9	9.2
6	Dry shoot weight (DSW)	8	RM22554(L)- RM8005(R)	3.83	5.81	1.5	10	5.5–1.01	4.49
7	Root volume (RV)	7	RM5847(L)-RM22031 (R)	3.05	14.33	-3.4	231	23.59–26.3	2.71
8	Root volume (RV)	8	RM22554(L)-RM8005(R)	4.68	14.24	3.39	14	5.5–1.01	4.49
9	Yield (SY)	8	RM22554(L)-RM8005(R)	5.5	18.5	1.76	10	5.5–1.01	4.49
10	Biomass	8	RM22554(L)-RM8005(R)	5.89	11.42	3.21	11	5.5–1.01	4.49
11	Biomass	1	WR 1.7(L)-RM8105(R)	3.1	15.44	3.73	40	1.7–10.9	9.2
12	Root/shoot ratio (RS ratio)	8	RM22554(L)-RM8005(R)	5.89	11.42	3.21	11	5.5–1.01	4.49
13	Root/shoot ratio (RS ratio)	1	WR 1.7(L)-RM8105(R)	3.1	15.44	3.73	40	1.7–10.9	9.2
14	P content of Seed	1	WR 1.7(L)-RM8105(R)	2.75	10.51	0.23	40	1.7–10.9	9.2
15	P content of Seed	7	RM21521(L)-RM21103(R)	2.87	10.01	-0.22	87	15.9–4.59	11.31
16	P content of Seed	8	RM22554(L)-RM8005(R)	2.75	2.25	3.21	9	5.5–1.01	4.49

### Epistatic interaction among the QTLs

A total of four significant epistatic interactions were observed for different component traits associated with tolerance to low soil P. The genomic region on chromosome 8, between the marker interval of RM22554 and RM8005 (which is a QTL hot-spot) showed epistatic interaction for the number of productive tillers with the region on chromosome 11, located between the marker interval of RM26784 and RM27096 and this interaction contributed to 31.82% of phenotypic variation. For the trait panicle length, epistatic interaction was observed between genomic region on chromosome 7, flanked by the marker, RM5720 and RM21521 with genomic region on chromosome 11, located between the marker interval of RM26550 and RM26784 with 20.31% phenotypic variation. Another epistatic interaction was observed between the genomic regions on chromosome 2 (flanked by marker interval, RM13709 and RMES-2-1) and the genomic region on chromosome 8 (flanked by the marker interval, RM8005 and SSR-8-19) for the trait, root volume, with a phenotypic variation of 27.83%. A fourth significant epistatic interaction observed for the traits biomass and root to shoot ratio between the genomic region on chromosome 8 (flanked by the marker interval, RM8005 and SSR-8-19) and the genomic region on chromosome 12 (flanked by the marker interval, RM28346 and RM5479) and this interaction contributed to 24.90% of the phenotypic variation (S2 Fig; Table 3).

**Table 3:** Epistatic interaction observed for various traits studied for low soil P tolerance.

Trait Name	Chr 1^a^	Position1 (cM)^c^	Flanking markers Chr 1^a^	Chr 2 ^a^	Position2 (cM) ^d^	Flanking markers Chr 2 ^a^	LOD	PVE (%)	Add^a^	Add^b^	Add byAd
Number of productive tillers	Chr 8	0	RM22554-RM8005	Chr 11	75	RM26784-RM27096	5.67	31.83	0.499	-0.049	1.24
Panicle length	Chr 7	10	RM-5720-RM21521	Chr 11	15	RM26550-RM26784	5.29	20.32	0.376	0.467	-1.95
Root volume	Chr 2	65	RM13709- RMES-2-1	Chr 8	40	RM8005-SSR-8-19	5.84	27.84	-1.272	2.114	-4.88
Biomass	Chr 8	40	RM8005-SSR-8-19	Chr 12	30	RM28346-RM5479	5.62	24.91	1.282	-0.376	-4.37
Root /shoot ratio	Chr 8	40	RM8005-SSR-8-19	Chr 12	30	RM28346-RM5479	5.62	24.91	1.282	-0.376	-4.37

**Note:** PVE%- phenotypic variation, Add- additive, LOD- logarithm of the odds, Add by Ad- additive by additive effect, Chr1^a^ - the first chromosome and QTL chromosomal interval, Chr2^a^ - the second chromosome and QTL chromosomal interval, Add^a^- additive effect of the first QTL involve in interaction, Add^b^- additive effect of the second QTL involve in interaction, c- position of first chromosome, d- position of second chromosome.

### *In-silico* analysis of the genomic region spanning the major QTLs for identification of putative candidate genes

*In-silico* identification of the genes present within the QTL interval on chromosome 1 (between the markers, WR 1.7 and RM 8105), revealed a total of 1339 genes encoding various biological functions. Among them, 14 genes related to either phosphorus uptake or its utilization were identified (S2 Table). Four of these genes are associated with auxin response and may be related to root and cell growth. Other genes encode proteins of important functions like acid phosphatase activity and related activities. A total of 10 QTLs were identified in the genomic region (between the marker interval of RM22554 and RM8005) flanking the QTL hotspot on chromosome 8. *In-silico* analysis of QTL hot-spot resulted in the identification of a total of 538 genes. Among them, eight genes were found to be related to P uptake or utilization, and others were related to genes encoding protein containing Myb, Zinc-finger domain, and acid phosphatase or vanadium-dependent haloperoxidase related family proteins (S3 Table).

## Discussion

Significant phenotypic variation has been observed in rice for different traits related to productivity under low soil P conditions, indicating the possibility of increasing rice production in P poor soils [18–20]. Breeders are attempting to develop rice cultivars tolerant to soil P-deficiency using the recently discovered and well characterized major QTL, *Pup1* [10, 21] and some success has been witnessed in this direction [12, 22, 23]. However, *Pup1* is the only major QTL associated with low soil P tolerance identified so far and there is an imminent need to identify additional, novel, non-*Pup1* type QTLs (and the genes underlying them) associated with low P tolerance in rice. It is expected that such efforts will provide scope for developing better low soil P tolerant varieties and possibly reduce application of P fertilizers in rice. Through an earlier study [3], we identified a novel rice line, named Wazuhophek, which exhibited excellent tolerance to low soil P levels and was devoid of *Pup1* (S3 Fig). We hypothesized that Wazuhophek might have novel mechanism(s) of tolerance to low soil P and may harbor novel genes/QTLs associated with tolerance, with potential utility in breeding. Hence, we developed a RIL population from Wazuhophek and used it for molecular mapping in the present study.

Even though the number of markers used in this study and their density was considerably lesser as compared to some previous studies [24, 25], we were successful in identifying at least seven major QTLs associated with different component traits of tolerance to low soil P, including yield under low soil P. This was possible due to intensive phenotyping carried out under natural field conditions coupled with molecular mapping using reasonably uniformly distributed set of rice SSR markers and deployment of a mapping population of optimal size.

Continuous distribution of phenotypic frequency of different traits associated with tolerance supports the quantitative inheritance of the traits associated with tolerance to low soil P in Wazuhophek. We observed that low soil P-deficiency resulted in reduced shoot growth based on low phenotypic values for traits like plant height, shoot dry weight, and total dry weight, similar to the earlier report [24] and also in some of our earlier studies related to low soil P tolerance [3, 26]. A near-normal platykurtic distribution observed for most of the traits analyzed in this study suggests that there is a high frequency of RILs with intermediate phenotype and hence the component traits associated with low soil P tolerance is indeed controlled by multiple loci (i.e. polygenic inheritance).

Similar to previous reports [8, 20, 27], in this study also, RILs with more productive tillers per plant showed enhanced P deficiency tolerance. A correlation analysis was performed to understand the nature of the relationship among the 14 quantitative characters, under low P soil. Most of the component traits were correlated with each other and the genetic loci controlling these traits were in close proximity as reported previously [9, 24, 28, 29]. Root dry weight and shoot dry weight showed relatively high correlation under low P condition and the number of productive tillers showed significant and positive association with root dry weight and shoot dry weight and biomass as reported earlier [27]. Root length was significantly correlated with root volume, root dry weight, and biomass, and plant height showed significantly positive association with root volume, root dry weight, and biomass. These results are as per the earlier report [24, 25]. Grain yield per plant displayed significant and positive association with root length and 1000 grain weight and similar observations were reported earlier [30] in rice under drought stress condition. Most of the tolerant RILs had more root elongation and more number of tillers compared to the other lines, as also noted in an earlier study [31].

RIL mapping populations, which are a series of homozygous lines, are the most commonly used populations for reliable QTL mapping [32] as plants within a line will not show any difference and are expected to be homogenous. Earlier studies have identified QTLs related to low soil P tolerance on chromosome 1 [25, 33]. In the present study a total of four co-located QTLs were detected on chromosome 1, out of which two were minor QTLs for the traits, dry shoot weight (*qDSW1*) (PVE-14.11%) and P content in seed (*qSP1*) (PVE-10.51) and the other two were major QTLs for the traits, total biomass (qBiomass1) (PVE-15.44%) and root to shoot ratio (qr1) (PVE-15.44%), located between the marker intervals of WR1.7- RM8105 (Table 2). A two-year field experiment under low P conditions has carried out and three yield associated and eight phosphorus use efficiency-related traits were investigated in this study and nine QTLs were identified for different traits, including a major one for PUE on chromosome 1 [33]. The QTL for biomass and P content of seed identified on chromosome 1 in this study is at a location which is different from that of the major QTL identified earlier [33]. In another study [25] backcross introgression lines (BILs) derived from an inter-specific cross (*Oryza sativa* L. and *O*. *rufipogon* Griff.) used for molecular mapping of P deficiency tolerance related traits and identified QTLs on chromosome 1 for relative root dry weight, relative shoot dry weight, relative root length, relative shoot fresh weight and relative total biomass dry weight. Additional QTLs on Chromosome 1 have been identified for P deficiency related traits like relative total dry weight and P-deficiency induced activity of acid phosphatase [24, 34]. However, all these QTLs identified earlier were mapped at locations on chromosome 1, which is different from the location of QTLs identified in this study. In earlier studies, QTLs responsible for traits associated with tolerance to low soil P conditions have been identified on chromosome 8 [25, 33]. Among them, one of the QTLs was associated with relative root dry weight with LOD score of 2.53 and it contributed for 6.13% of phenotypic variation [25]. Further, two QTLs were identified on Chromosome 8 for P translocation and P translocation efficiency and they contributing for 5% and 7.5% of phenotypic variation, respectively [33]. However, none of the earlier identified QTLs on chromosome 8 are at location similar to the QTL hotspot identified on Chr. 8 in this study. Hence, the QTLs identified in the hotspot in our study can be considered novel and they will certainly be useful in breeding for low soil P tolerance, as the LOD and PVE values are considerably higher as compared to those identified in earlier studies.

Epistasis is defined as the deviation from the sum of the independent effect of individual genes controlling quantitative traits. It is a non-allelic interaction that may change the magnitude of the phenotypic expression of the QTLs by suppressing/enhancing the expression of the interacting genes [35, 36]. In the current study, negative epistatic interactions were observed for panicle length, root volume, biomass and root to shoot ratio, whereas number of productive tillers exhibited positive epistatic interaction (Table 3). Very few epistatic interactions have been observed in the present study, which may be due to background noise interference. Four out of five epistatic interactions were specific for the genomic region located on Chromosome 8 flanked by the markers RM22554, RM8005, and SSR-8-19, which indicates the active involvement of this hotspot region with low P soil deficiency tolerance (Table 3; S2 Fig).

In order to identify key putatively expressed candidate genes in the genomic region spanning the major QTLs, we carried out *in silico* analysis. We identified some genes of interest and they may be possibly related to P uptake and utilization in rice. These genes include genes related to auxin responsive activity, acid phosphatase activity, protein with the SPX domain, WD40 protein and SHR transcription factors which contribute to morphological and physiological changes in plants involved in low soil P tolerance which also reported in earlier studies [37–40].These genes need to be cloned and further to draw any meaningful conclusions about their possible role in low soil P tolerance in Wazuhophek. In previous study Nagina22 mutants screened under low P and alternative wet and dry condition to identify marker associated with yield traits under these conditions and the selected mutants and associated markers identified in this study can be used for breeding programme and gene discovery for low soil P tolerance and alternative wet and dry condition [41]. In conclusion, this study has identified seven major and nine minor effect QTLs and a major QTL hotspot on chromosome 8 for low soil P tolerance from the donor, Wazuhophek and they are different from the QTLs associated with the trait discovered in earlier studies. Fine-structure mapping of the major QTL hot-spot region identified in this study using high-throughput SNP markers coupled with *in-silico* candidate gene identification in the fine-mapped region and their analysis and characterization using QTL-NILs will offer further insights into the molecular mechanisms associated with the tolerance to low soil P in Wazuhophek. Further, the major QTLs/QTL hot-spot region identified from the present study on chromosome 8 can be transferred into elite rice varieties, which are sensitive to low soil P levels, through marker-assisted breeding along with the other major QTL, *Pup1* to enhance the level of tolerance and to improve P-use-efficiency. In addition RILs with novel QTL for low P tolerance can also be used in various crossing programs.

## Supporting information

S1 Fig Linkage map and the position of identified QTL for low soil P tolerance. (TIF)

S2 Fig Epistatic interaction observed for various traits studied for low soil P tolerance. NPT- number of productive tillers, PL- panicle length, RV- root volume, root/shoot- root to shoot ratio.
(TIF)

S3 Fig Haplotype information of chromosome 12 in respective to *Pup1*/non *Pup1* allele. A:- chromosomal region of chromosome 12 other than *Pup1* allele, K:- Kasalath specific *Pup1* allele, N:- Improved samba Mahsuri/ Wazuhophek specific non *Pup1* allele.
(TIF)

S1 Table Descriptive statistical data of yield and yield related traits obtained under low soil P condition. (DOC)

S2 Table List of annotated genes present within QTL intervals on chromosome 1, related to phosphorus utilization and uptake. (DOC)

S3 Table List of annotated genes present within QTL intervals on chromosome 8, related to phosphorus utilization and uptake. (DOC)
