# Supplementary figures and images for: Novel major QTLs associated with low soil phosphorus tolerance identified from the Indian rice landrace, Wazuhophek
Source: PLoS One. 2021 Jul 15;16(7):e0254526. doi: 10.1371/journal.pone.0254526 (PMC8282084; doi:10.1371/journal.pone.0254526)

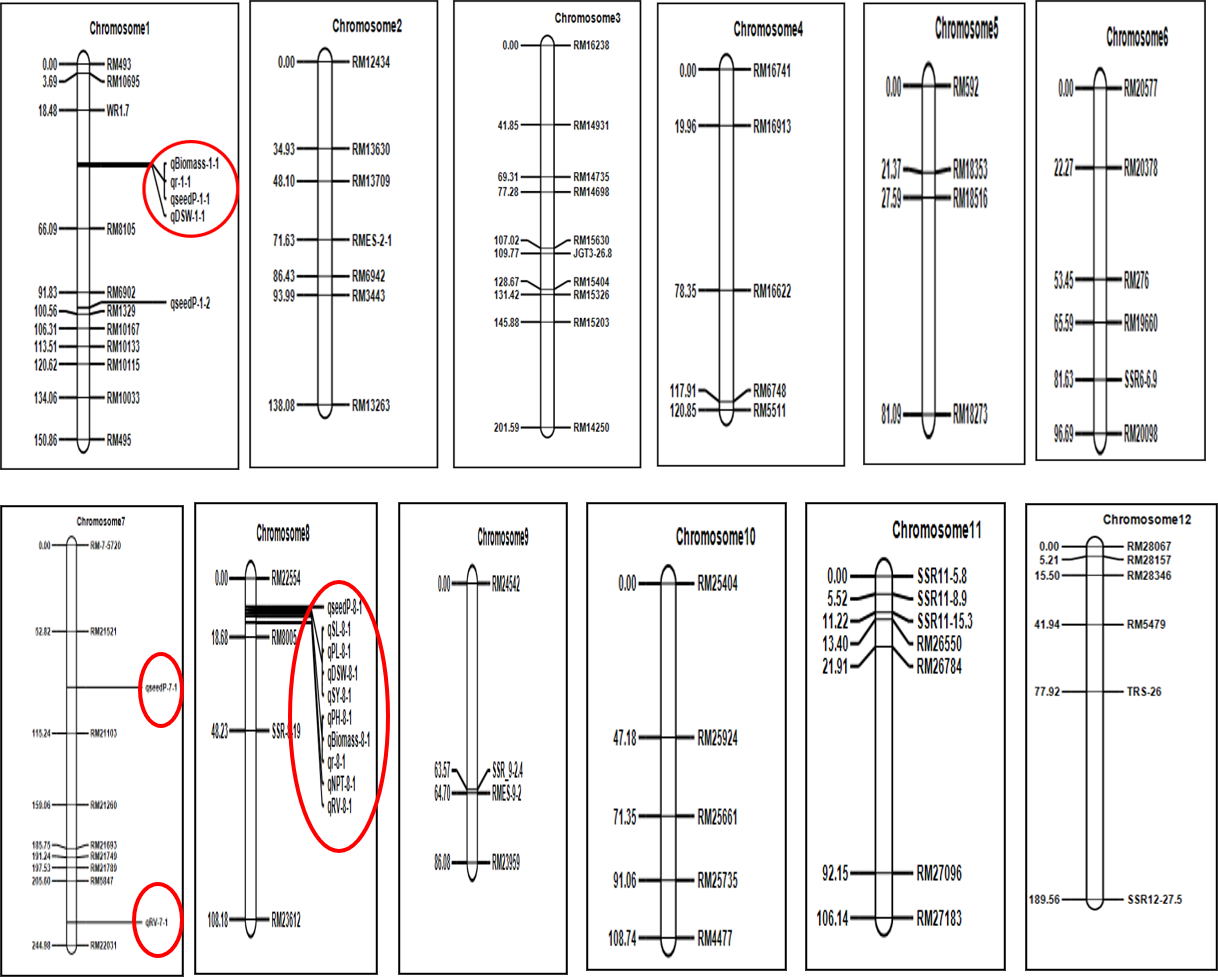

Supplement: S1 Fig — (TIF) [file pone.0254526.s001.tif]

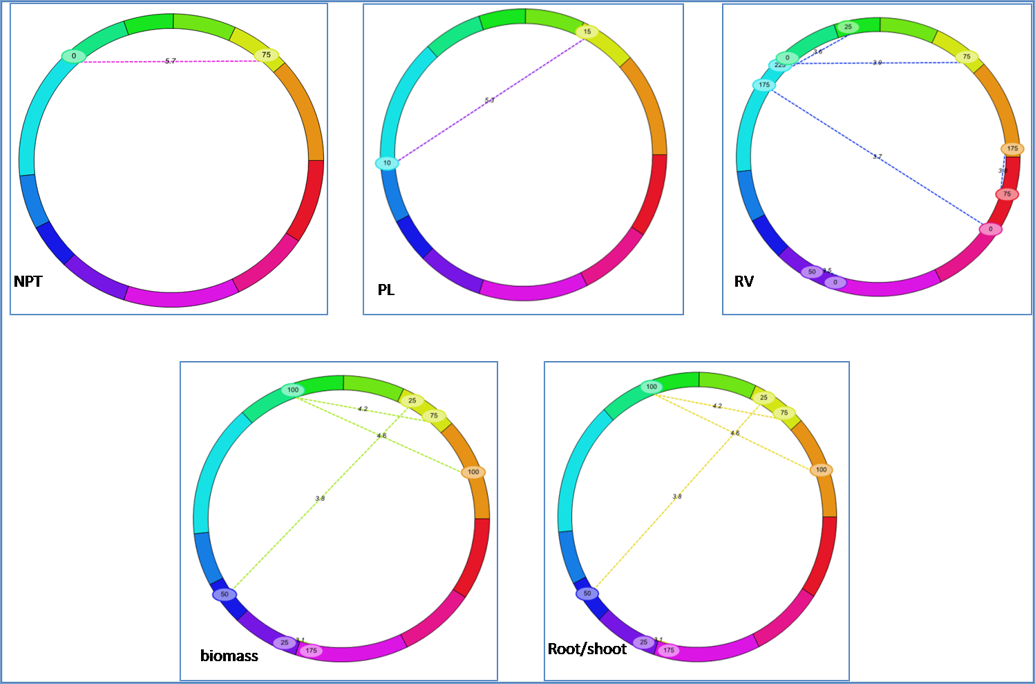

Supplement: S2 Fig — NPT- number of productive tillers, PL- panicle length, RV- root volume, root/shoot- root to shoot ratio. (TIF) [file pone.0254526.s002.tif]

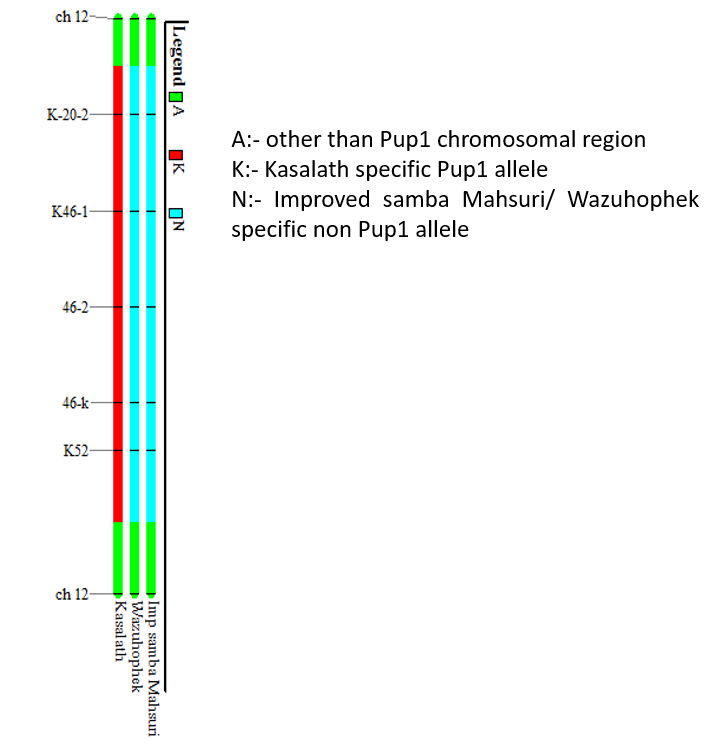

Supplement: S3 Fig — A:- chromosomal region of chromosome 12 other than Pup1 allele, K:- Kasalath specific Pup1 allele, N:- Improved samba Mahsuri/ Wazuhophek specific non Pup1 allele. (TIF) [file pone.0254526.s003.tif]
