# Supplementary material for: Novel major QTLs associated with low soil phosphorus tolerance identified from the Indian rice landrace, Wazuhophek
Source: PLoS One. 2021 Jul 15;16(7):e0254526. doi: 10.1371/journal.pone.0254526 (PMC8282084; doi:10.1371/journal.pone.0254526)
Supplement: S1 Table — (DOC) [file pone.0254526.s004.doc]

**Supplementary Table 1: Descriptive statistical data of yield and yield related traits obtained under low soil P condition**

| **SI.**  **No** | **Character** | **Wazuhophek** | **ISM** | **RIL’s** | | | | | | |
| --- | --- | --- | --- | --- | --- | --- | --- | --- | --- | --- |
|  |  |  |  | **Mean** | **SEd** | **Min** | **Max** | **% trans segregates** | **Skewness** | **Kurtosis** |
| 1 | Plant height (cm) | 73.0 | 51.0 | 69.9 | 11.8 | 40.0 | 96.33 | 42.1 | 0.02 | -0.3 |
| 2 | Shoot length (cm) | 68.0 | 48.0 | 61.2 | 12.0 | 37.0 | 89.0 | 43.8 | -0.02 | -0.4 |
| 3 | Number of productive tillers/plant | 8.0 | 2.0 | 4.21 | 1.7 | 1.0 | 9.0 | 44.7 | 0.3 | -0.3 |
| 4 | Panicle length (cm) | 18.0 | 11.0 | 14.2 | 3.1 | 7.0 | 22.6 | 20.1 | -0.08 | -0.1 |
| 5 | Root length (cm) | 26.0 | 22.0 | 23.2 | 3.7 | 14.0 | 32.5 | 50.0 | -0.2 | -0.04 |
| 6 | Dry shoot weight (gm) | 5.2 | 2.0 | 6.30 | 3.2 | 0.9 | 13.3 | 67.5 | 0.2 | -0.9 |
| 7 | Dry root weight (g) | 2.5 | 0.2 | 1.21 | 0.8 | 0.1 | 6.4 | 8.7 | 2.4 | 10.9 |
| 8 | Root volume (ml) | 20.0 | 10.0 | 15.3 | 7.09 | 5.0 | 30.0 | 37.7 | 0.5 | -0.7 |
| 9 | Root shoot ratio | 0.4 | 0.1 | 0.19 | 0.09 | 0.05 | 0.7 | 5.2 | 3.3 | 15.7 |
| 10 | Grain yield per plant (g) | 9.0 | 2.5 | 3.9 | 3.06 | 0.1 | 11.4 | 50.8 | 0.6 | -0.6 |
| 11 | 1000 seed weight (g) | 22.1 | 11.0 | 16.6 | 2.8 | 10.0 | 27.2 | 35.9 | 3 | 0.9 |
| 12 | Total biomass (g) | 14.2 | 4.5 | 10.2 | 5.5 | 1.06 | 22.8 | 42.1 | 0.2 | -0.9 |
| 13 | Days to 50 per cent flowering (Days) | 134.0 | 122.0 | 126.6 | 2.9 | 119.0 | 134.0 | 7.8 | -0.4 | 0.002 |
| 14 | P content in grains (mg g^-1^**)** | 0.5 | 0.09 | 0.45 | 0.3 | 0.08 | 1.1 | 56.5 | 0.14 | -0.9 |
