# Supplementary material for: Novel major QTLs associated with low soil phosphorus tolerance identified from the Indian rice landrace, Wazuhophek
Source: PLoS One. 2021 Jul 15;16(7):e0254526. doi: 10.1371/journal.pone.0254526 (PMC8282084; doi:10.1371/journal.pone.0254526)
Supplement: S2 Table — (DOC) [file pone.0254526.s005.doc]

**Supplementary Table 2: List of annotated genes present within QTL intervals on chromosome 1 , related to phosphorus utilization and uptake**

| **Locus ID** | **Position (bp) (start-end)** | **No. of Exons** | **Description (RAP-DB annotation)** |
| --- | --- | --- | --- |
|
| Os01t0127200-01 | 1,497,241-1,500,606 | 4 | Multicopper oxidase, Maintenance of Pi homeostasis |
| Os01t0138600-01 | 2,063,615..2,065,995 | 1 | phosphotransferase system, PEP-utilising enzyme, N-terminal domain containing protein |
| Os01t0139600-01 | 2,097,192-2,100,692 | 9 | Similar to Lipid phosphate phosphatase 2 (EC 3.1.3.-) (AtLPP2) (Phosphatidic acid phosphatase 2) (AtPAP2) (Prenyl diphosphate phosphatase) |
| Os01t0165000-01 | 3,356,383-3,358,426 | 2 | Transcription factor, Dehydration and salt stress tolerance |
| Os01t0169800-01 | 3,577,473-3,581,859 | 5 | Tryptophan aminotransferase, Indole-3-acetic acid (IAA) biosynthesis |
| Os01t0172100-01 | 3,711,212-3,713,653 | 9 | Similar to Triose phosphate/phosphate translocator, non-green plastid, chloroplast precursor (CTPT) |
| Os01t0178500-02 | 4,073,916-4,076,438 | 5 | A member of rice Aux/IAA family, Cross-talk of auxin and brassinosteroid signaling pathways, Plant morphogenesis |
| Os01t0191200-02 | 4,872,158-4,873,594 | 2 | Similar to Acid phosphatase |
| Os01t0191700-01 | 4,905,790-4,909,119 | 1 | Similar to Pyrophosphate-fructose-6-phosphate 1-phosphotransferase-like protein (Pyrophosphate-dependent phosphofructo-1-kinase-like protein) |
| Os01t0231000-03 | 7,249,868-7,253,703 | 5 | Similar to Auxin-responsive protein (Aux/IAA) (Fragment). |
| Os01t0236300-01 | 7,547,017-7,551,842 | 14 | Similar to Auxin response factor 18 |
| Os01t0239000-02 | 7,686,901-7,693,781 | 6 | Similar to Phosphate starvation regulator protein (Regulatory protein of P- starvation acclimation response Psr1) |
| Os01t0239200-01 | 7,718,270-7,722,217 | 12 | Similar to Phosphate translocator (Fragment) |
| Os01t0259600-01 | 8,701,299..8,708,612 | 1 | Similar to phosphoadenosine phosphosulfate (PAPS) reductase family protein |
