# Supplementary material for: Novel major QTLs associated with low soil phosphorus tolerance identified from the Indian rice landrace, Wazuhophek
Source: PLoS One. 2021 Jul 15;16(7):e0254526. doi: 10.1371/journal.pone.0254526 (PMC8282084; doi:10.1371/journal.pone.0254526)
Supplement: S3 Table — (DOC) [file pone.0254526.s006.doc]

**Supplementary Table 3: List of annotated genes present within QTL intervals on chromosome 8, related to phosphorus utilization and uptake**

| **Locus ID** | **Position (bp) (start-end)** | **No. of Exons** | **Description** |
| --- | --- | --- | --- |
|
| Os08t0118500-00 | 1,039,566-1,040,187 | 1 | Similar to Auxin-induced SAUR-like protein |
| Os08t0118800-00 | 1,045,709-1,046,017 | 1 | Similar to SAUR31 - auxin-responsive SAUR family member |
| Os08t0122000-01 | 1,205,886-1,209,749 | 3 | Similar to Protein phosphatase 2A B' regulatory subunit |
| Os08t0127500-01 | 1,578,268-1,580,227 | 5 | Acid phosphatase/vanadium-dependent haloperoxidase related family protein |
| Os08t0135100-01 | 1,994,522-1,998,857 | 8 | Similar to Phosphate/phosphoenolpyruvate translocator protein-like |
| Os08t0138700-01 | 2,159,517-2,162,918 | 7 | Serine/threonine protein kinase-related domain containing protein |
| Os08t0144000-01 | 2,433,858-2,436,741 | 3 | Myb, DNA-binding domain containing protein |
| Os08t0151700-01 | 2,972,601-2,976,414 | 9 | Zinc finger, RING/FYVE/PHD-type domain containing protein |
